# A Historical Review of Our Knowledge of Brown Lemming Population Cycles at Barrow, Alaska: Cycles No More or Never Before

**DOI:** 10.3390/ani15233436

**Published:** 2025-11-28

**Authors:** Denver W. Holt

**Affiliations:** Owl Research Institute, P.O. Box 39, Charlo, MT 59824, USA; owlmontana@blackfoot.net; Tel.: +1-406-644-3412

**Keywords:** Alaska, Barrow, brown lemming, collared lemming, cycles, density, movements, indicator species, population fluctuations

## Abstract

The literature for brown lemming (*Lemmus trimucronatus*) and collared lemming (*Dicrostonyx groenlandicus*) population cycles was revisited from Barrow, Alaska. This review covered observations and research primarily from 1946 to 1974. Much of what we know about brown lemming cycles from North America was derived from these early studies. The data for collared lemming, however, are far less extensive and only a minor part of the historical research at Barrow. Nonetheless, important information was discovered. Collectively, the historical literature is confusing and sometimes contradictory. The time intervals, amplitude, and density of lemming populations from Barrow varied greatly from year to year. For example, in most papers, 1956 was considered a lemming population high, but in a major research paper in 1993, the 1956 data was sometimes included and sometimes deleted because it did not meet an arbitrary mathematical definition of a population high. Qualitative explanations were often used to support the lemming population cycle concept when it was apparently in flux or did not exist. Other investigations suggested synchronous lemming population fluctuations over wide geographic areas did not occur, but rather were localized most of the time. Even within a specific local area, lemming densities varied with habitat. Presumably, higher densities were in higher quality habitats, but this could vary somewhat with season. It is unlikely that lemming migrations occurred; however, local movements of large numbers of lemmings were witnessed. Although many studies suggested a specific event influenced lemming population fluctuations, overall, the data suggest multiple factors acting synergistically drove the x-fold increases and x-fold decreases in lemming populations at Barrow. Other qualitative observations, and quantitative studies suggest lemming population fluctuations affect and have an effect on the survival and reproduction of other species of birds and mammals at Barrow. Brown lemmings should be considered an indicator of the health of the Arctic environment at Barrow. Clearly, population fluctuations of lemmings at Barrow existed with an average interval for peak populations of about 3.8 years, ranging from 2 to 6 years, depending on what data was included and how it was analyzed. The conundrum, however, is defining a peak. Furthermore, one must ask if an average interval between peak lemming populations is really a cycle. The data support population fluctuations; however, the four annual phases of the cycle (e.g., increase, peak, decline, low) did not repeat themselves in sequence. Overall, perhaps cycles did not exist in the strictest definition of the word at Barrow. Perhaps it is time to redefine Barrow lemming “cycles” as annual population fluctuations that exhibit patterns over time.

## Simple Summary

Plague-like population outbreaks of lemmings and voles have been known for centuries. And Arctic lemming population outbreaks have been observed since native people lived there. These observations became common knowledge and were verbally passed along from generation to generation. Written records of lemming outbreaks, and an interest by naturalists in why outbreaks occur, date back over a century. As standardized measures to study lemming species were developed, it was clear that outbreaks occurred only periodically (4–6 years), but patterns did emerge. Thus the concept of “cycle” was born. Whether the word “cycle” is the best descriptor or not is debatable. Nonetheless, the modern study of “cycles” began and continues today mostly using snap-trap and grid sampling methods. Both methods have strengths and weaknesses, and which method to use depends on the question(s). After decades of study, answers to what drives “cycles” remain confusing. Food, social strife, predation, disease, and so forth are known to affect outbreaks; however, no single factor appears to drive the “cycles”. Indeed, multiple factors acting independently and collectively appear to drive the outbreaks, which are not true mathematical cycles and are not predictable. Perhaps it is best to describe these “cycles” as population fluctuations that show patterns over time.

## 1. Introduction

For thousands of years, Arctic people have been aware of lemmings and lemming population fluctuations. Even in the past several hundred years, stories of lemming population outbreaks witnessed by Arctic explorers and residents living in Arctic landscapes are sprinkled throughout the literature. Although qualitative, these observations provide a historical record. Most accounts note that occasionally unusually high numbers of lemmings occur, and they are seen scurrying about the tundra in plague-like over-abundance. These anecdotes and stories have been nicely summarized in a few books and papers (Elton 1942 [1], Tamarin 1985 [2], Stenseth and Ims 1993 [3], Chitty 1996 [4], Krebs 2013 [5]).

Population fluctuations of small rodents have been studied for over 125 years (Collet 1895 [6]). And scientific research into small mammal life history provides clear quantitative results that prove extreme population highs followed by desolate population lows do occur. These highs and lows are often referred to as peaks and crashes and occur periodically over time—hence the term cycle.

It was Collet (1895 [6]) who perhaps founded the cycle concept. Later, the first major syntheses and historical overview of small mammal population fluctuations, outbreaks, and plagues was provided by Elton (1942 [1]). The cycle concept gained momentum as fur sale records of trapped animals such as lynx (*Lynx canadensis*), snowshoe hare (*Lepus americanus*), Arctic fox (*Vulpes lagopus*), and muskrat (*Ondatra zibethicus*) also showed an apparent cyclic occurrence (see Chitty 1996 [4]). Even some game bird and Snowy Owl (*Bubo scandiacus*) numbers were suggested to be cyclic (see Shelford 1945 [7], Gross 1947 [8], Chitty 1996 [4]). The cycle concept was further buttressed in North American lemming populations studies at Churchill, Manitoba, Canada (Shelford 1943 [9], 1945 [7]), Barrow, Alaska, United States (Pitelka 1957 [10,11], Baker Lake, Northwest Territories, Canada) Krebs 1964 [12]), and Krebs and Meyers 1974 [13].

Scientific and folklore explanations for lemming and vole population highs and lows are as old as the observations themselves (Elton 1942 [1], Tamarin 1985 [2], Stenseth and Ims 1993 [3], Chitty 1996 [4], Krebs 2013 [5]). For example, an interesting folklore suggests that lemmings march to the sea and commit mass suicide when under duress due to overpopulation (Elton 1942 [1], Stenseth and Ims 1993 [3], Chitty [4]).

However, research has shown that lemmings do not commit mass suicide, despite some die-offs during population declines. See Elton (1942 [1]) and Stenseth and Ims (1993 [3]) for excellent chronological overviews of the emergence of scientific study into lemming life history in Europe and North America.

Despite an enormous amount of research, no singular answer to lemming population fluctuations exists (see Taitt and Krebs 1985 [14], Stenseth and Ims 1993 [3], Krebs 1993 [15], Chitty 1996 [4], Pitelka and Batzli 2007 [16], 2018 [17], Krebs 2013 [5], Ehrich et al., 2020 [18], Gauthier et al., 2024 [19], Krebs 2024 [20]). Indeed, Krebs (2013 [5], 2024 [20]) concluded that multifactorial intrinsic and extrinsic factors, not singular events, drive lemming population fluctuations. But how all these factors interact is still under study.

In Alaska, where I conduct my research, lemming taxonomy has been in flux for a long time (Abramson 2001 [21], Jarrell and Fredga 1993 [22], Musser and Carleton 2005 [23], MacDonald and Cook 2009 [24]). For example, Jarrell (1994 [25]) recognized four species of *Dicrostonyx* and one species of *Lemmus*. Later, MacDonald and Cook (2009 [24]) recognized one species of *Dicrostonyx* and maintained recognition of the only *Lemmus* species for Alaska. A recent worldwide taxonomic assessment of lemmings now recognizes 16 species within four genera (*Dicrostonyx*, *Lemmus*, *Myopus*, and *Synaptomys*) within the Family Cricetidae (Llobet et al., p. 22, 2023 [26]). In this new assessment, two species of *Lemmus* and three species of *Dicrostonyx* are recognized in Alaska. For the Barrow region on the North Slope of Alaska, the Nearctic brown lemming (*Lemmus trimucronatus*) and Nearctic collared lemming (*Dicrostonyx groenlandicus*) are now the only species of lemmings recognized (Llobet et al., pp. 222–223, 2023 [26]).

Lemmings are a unique group of rodents adapted to habitats of northern Temperate and Arctic latitudes. Unfortunately, Arctic lemmings are difficult to study due to their distribution in remote northern latitudes, logistical constraints, weather variables, and their population fluctuations are not predictable. Nonetheless, Arctic lemmings are the most famous species of lemmings and are known to show dramatic population fluctuations—often referred to as cycles. Despite difficulties studying lemmings, in Canada and United States, *Dicrostonyx* has been the subject of several studies, yet much of what we know about *Lemmus* originally came from one long-term study on population demographics, cycles, and trends from Barrow, Alaska (Pitelka 1973 [27], Pitelka and Batzli 1993 [28], Pitelka and Batzli 2007 [16], 2018 [17]). Other studies from Canada that include *Lemmus* do exist, however (Krebs 1964 [12], Gruyer et al., 2008 [29], Gruyer et al., 2010 [30], Krebs et al., 2011 [31], 2011 [32], 2013 [5], Fauteux et al., 2015 [33], Gauthier et al., 2024 [19]). In fact, many of the above studies from Canada derive from one long-term project overseen by G. Gauthier. Also see Krebs (2013 [5]), Ehrich et al. (2020 [18]), and Gauthier et al. (2024 [19]) for reviews and analyses of long-term Arctic lemming research and monitoring of populations worldwide.

Finally, an important trait of Arctic lemming life history is the ability to breed under snow during late winter in certain years. However, studying lemmings when the ground is snow-covered has proved almost impossible (Thompson 1955 [34], Bilodeau et al., 2013 [35]) and consequently little quantitative data exists on what influences winter breeding. Nonetheless, efforts are being made (Soininen et al., 2015 [36]).

## 2. Study Area

The village of Barrow, Alaska (71°18′ N; 156°40′ W), is located on the Arctic Coastal Plain and is the most northerly point in Alaska. Topographic relief is low, from sea level to ~10 m, and ice-wedge-created polygonal ground, shallow lakes, and underlying permafrost dominate the landscape. Barrow is bordered by the Chukchi Sea to the west and the Beaufort Sea to the east (see Brown et al., 1980 [37] for a habitat overview). This area has also been described as the Beaufort Coastal Plain, an eco-region stretching from western Canada across Alaska, and bordering the Arctic Ocean, basically north of the Brooks Mountain Range (Nowacki et al., 2001 [38]).

## 3. Justification for a Review

Aside from a few historical observations at Barrow (Murdock 1885 [39], Stone 1900 [40], Brower 1942 [41], Rausch 1950 [42], Vaughn 1992 [43]), numerous ecological studies of birds and mammals were conducted from about 1946 to 1974. Within these years, I concentrated mostly on Frank A. Pitelka’s data from 1955 to 1974. In this review, 28 studies are direct results of Pitelka. At least six other publications have older historical-related information, while five newer studies provide data collected within the last 25 years, independent of Pitelka. Of particular note for this review of lemming natural history and lemming cycles at Barrow, include the following: Rausch 1950 [42], Thompson 1955 [34,44], Bee and Hall 1956 [45], Pitelka 1957 [10,11], Pitelka 1972 [46], Pitelka 1973 [27], Batzli 1975 [47], Batzli et al., 1980 [48], Batzli 1981 [49], Batzli et al., 1983 [50], Pitelka and Batzli 1993 [28], Pitelka and Batzli 2007 [16], Pitelka and Batzli 2018 [17].

After 1974, no other research on lemmings at Barrow was conducted until I began a study of the predator–prey relationship between Snowy Owls and brown and collared lemmings in 1992. My initial question was to determine if Snowy Owls were cyclic, and in pace with purported lemming cycles at Barrow (Pitelka and Batzli 1993 [28]). Thus, my colleagues and I established a lemming snap-trap program similar to, but not a replicate of, Pitelka and colleagues’ research. The Snowy Owl study is now in its 35th year, while the lemming snap-trap study is now in its 33rd year, all within a 214 km^2^ study area (see Holt et al., 2024 [51], Holt et al., 2025 [52]).

The lemming story at Barrow is very complicated. Given my three decades of field study, the concept of predictable Snowy Owl and lemming cycles did not materialize. Yet, clear population fluctuations and patterns did emerge. Many local native Iñupiat Eskimo residents and wildlife researchers believe lemming cycles at Barrow have collapsed or are out of synchrony due to climate change. Is the answer that simple?

Consequently, I felt it prudent to reread and objectively review the history of lemming and other lemming-related research at Barrow. For example, there were differing conclusions reached by other researchers in opposition to Pitelka and Batzli’s many qualifying statements that support the cycle concept. And qualitative and quantitative observations by native Iñupiat people and other researchers from Barrow suggested that, when lemmings reach peak populations, many other species of birds and mammals have higher breeding outputs. On the contrary, when lemming populations decline, breeding output of these other species also declines, as predation rates increase on these species.

In 2007, 14 years after their last research paper, Pitelka and Batzli (2007 [16]) published a historical review of their lemming research at Barrow. Although the review was informative, it was not a true independent review as the authors assessed their own research. Furthermore, the authors did not review the relationship between lemmings and other wildlife species. Thus, I believe a true independent historical review of lemming cycles at Barrow is a reasonable and justifiable proposition and has not been done before.

## 4. Objectives of This Review

This review is timely because of recent interest in worldwide lemming population responses to climate change. My specific objectives are as follows: (1) define the etymology/origin of the word cycle; (2) define cycle and associated terms from an ecologist’s perspective; (3) review the historical chronology of literature related to lemming research at Barrow; (4) review lemming life history traits associated with cycles at Barrow and compare these data with relevant literature; (5) determine if qualitative and quantitative evidence suggests an association between lemmings and other wildlife species at Barrow; (6) determine if qualitative and quantitative evidence suggests if lemmings are an indicator species for birds and mammals at Barrow; and (7) suggest future research questions for Barrow.

I followed Merriam-Webster’s online dictionary: https://www.merriam-webster.com (accessed on 19 November 2025 [53] to define the English word cycle). The Merriam-Webster Dictionary has been used to define words in the English language since 1828.

### 4.1. Define the Etymology/Origin of the Word Cycle

The word cycle is believed to derive from the Latin word *cyclus* and the Greek word *kyklos*, meaning a circle or wheel. According to the Merriam-Webster On-line Dictionary (www.Merriam-Webster.com (accessed on 19 November 2025) a cycle is defined as follows: (1) an interval of time during which a sequence of a recurring succession of events or phenomena is completed (a 4-year cycle of growth and development); (2) a course or series or events or operations that recur regularly and usually lead back to the starting point.

### 4.2. Define Cycle and Associated Terms from an Ecologist’s Perspective

The following definitions have been modified by many authors in various books and papers to define the ecology of animals, such as animal cycles. I use these definitions as they relate primarily to small mammals. Defining a cycle is complicated. For example, the moon rotates around the earth about 29.5 days. The earth rotates around the sun for about 365 days. These are examples of somewhat predictable and evenly spaced mathematical patterns, and we expect them to occur with regularity, cyclic if you will—in the purest definition of the word. However, using this definition for other natural events is less convincing. For example, animal cycles, drought cycles, extinction cycles, fire cycles, glacial cycles, hurricane cycles, ice sheet rotation cycles, ocean current cycles, sunspot cycles, and so on.

In wildlife population ecology, we have faithfully accepted the concept of animal cycles (e.g., boreal seed-eating birds, grouse, hares, lemmings, lynx, muskrats, voles, etc.). Clearly, some species such as lemmings and voles experience periodic spring and summer population highs, followed by multiple years of population lows. The question related to cycles, however, lies in the regularity or variability of intervals between population peaks, the annual amplitude variation, and the annual density variation per unit of measure (e.g., ha or ac) versus letting each year stand independently.

In the following paragraphs, I provide a brief historical overview of small mammal cycle definitions. In reviewing studies of *Microtus* vole cycles, Taitt and Krebs (1985 [14]) defined two categories of cycles: (1) annual fluctuations and (2) multi-annual cycles. For annual population fluctuations, there is usually a <5-fold increase in density, whereas for cyclic population fluctuations, there is usually a >10-fold increase in density. Taitt and Krebs (1985 [14]) concluded that annual population fluctuations reach maximum densities typically one-third of cyclic densities. And a characteristic of annual population fluctuations was a substantial spring decline in both sexes, whereas a characteristic of multi-annual cycles was a spring decline sometimes confined to males. The authors did acknowledge shortcomings in these definitions.

Later, Krebs (1996 [54]) revisited the cyclic definition issue. He felt there was little harm in using the word cycle to explain periodic fluctuations of mammal populations, as long as we realize these are not “true mathematical cycles”. He went on to state that periodic fluctuations recur with some regularity. Stenseth and Ims (1993 [55]) defined a lemming cycle as “a pattern of periodic fluctuations in the size of the population with rather constant period (the time between successive peaks), but possibly with highly variable amplitude.” Later, Krebs (2011 [56]) and Krebs (p. 192, 2013 [5]) moved towards calling cycles population fluctuations. Pitelka and Batzli (p. 218, 1993 [28]) defined multi-annual cycle of Microtine rodents at their Barrow, Alaska, study sites as “periodic episodes of high population density followed by very low population density (the extremes in density spanning more than a ten-fold difference).” In the High Arctic of Canada, a peak lemming cycle was defined as 10 lemmings/ha or 3/per/100-trap-nights, or when the density of a species is higher in one year than the proceeding or following year (Krebs et al., 2002 [57]).

From a more theoretical standpoint during the 1980s, researchers from Fennoscandia countries defined cycles using mathematical models that analyze periodicity (s), whereas if s > 0.50, the population is cyclic; if s < 0.50, the population is not cyclic (Henttonen et al., 1985 [58]).

## 5. Historical Chronology of Lemming Cycles at Barrow

The Barrow region has been occupied by various Eskimo people for 1200–1500 years (Brown et al., 1980 [37]) or around 4000 years (Ann Jensen, pers. comm). Survival on both land and sea was dependent on knowledge of natural resources, such as wildlife (see Norton 2001 [59]). In the Barrow region, two species of lemmings occur—brown lemming and collared lemming—and they are the only species of small rodents in the Barrow region. The Iñupiat people of Barrow have long recognized these two lemming species, and that periodic population fluctuations occur—particularly among brown lemmings (Brower 1942 [41], Bee and Hall 1956 [45], Rausch 1950 [42], Rausch 2001 [60]). The Iñupiat people call the brown lemming aviŋŋaq and the collared lemming qilaŋmiutauraq. Because the brown lemming was the focus of almost all research, unless otherwise noted herein, the lemming cycles at Barrow refer primarily to brown lemmings.

John Murdock provided one of the first written descriptions of brown lemming population fluctuations in the Arctic. In 1882 at Barrow, he wrote that there were no lemmings and few Snowy Owls. In 1883, he wrote, lemmings were “exceedingly plenty” and Snowy Owls proportionately abundant (Murdock, p. 102, 1885 [39], also in Bent 1938 [61], and Rausch 2001 [60]). In 1897–1898, E.A. McIlhenny described a population high of lemmings at Barrow (Stone 1900 [40], also in Pitelka 1957 [10]). These notes remain two of the earliest written documentations of brown lemming population highs at Barrow. In 1888, Eskimo people told Charles D. Brower (Brower, p. 123, 1942 [41]) that when lemmings were abundant, Snowy Owls and jaegers were also more abundant, and this happens only every few years. Also see Vaughn (1992 [43]) for a review of early explorers, naturalists, and whaling expeditions to the Arctic.

In 1948 and 1949, Weber (1950 [62]) noted that lemmings destroyed so much vegetation that their populations declined before snow melt. He attributed this decline to starvation. Around the same time, in 1949, Rausch (1950 [42]) witnessed a lemming population high in late May, and rapid decline as snow melted. Few lemmings were seen by early June (Rausch 1950 [42]). Rausch then published perhaps the first “scientific” paper on a brown lemming population high and decline at Barrow. In this publication, Rausch included a verbal communication from a “very reliable Eskimo” who told him 1946 was also a high lemming year. Thus, lemming population highs were recorded in 1946 and 1949.

In 1951 and 1952, an effort was made to catalog species of mammals on Alaska’s North Slope, determine their distribution, and describe their natural history (Bee and Hall 1956 [45]). To achieve this, Bee and Hall interviewed native people, reviewed historical records, cited primary literature, and traveled throughout the North Slope to collect hundreds of specimens. These chapters—particularly for brown lemmings—describe lemming systematics, distribution, habitat, skeletal measurements, behavior, body mass, sex ratios, reproductive biology, pelage development, nest chambers, factors affecting lemming life history, and population cycles.

Bee and Hall suggested both species of lemmings were relatively uncommon below 70° N and numerically increased from south to north. The greatest abundance of lemmings was on the Arctic Coastal Plain, particularly near Barrow. Here, brown lemmings reached their highest concentrations (Bee and Hall 1956 [45]). In fact, Bee and Hall (1956 [45]) made enormous contributions to our understanding of lemming biology from Barrow. They even suggested how cycles developed. Yet they received very little recognition for their work. Why their data were not cited in other research studies from Barrow is puzzling. Were they ignored?

Concurrently, the first organized study of lemmings in Barrow began and was conducted from 1950 to 1954 (Thompson 1955 [34,44]). Here, Thompson investigated a wide range of lemming life history traits, including food and predation. Thompson (1955 [34]) concluded that the 1949 population high and rapid decline was a “mass die-off”. However, by 1953, the lemming population was high again, and lemmings appeared to be everywhere as mass movements were occurring (Thompson 1955 [34]). By August 1954, the population was low again (Thompson 1955 [34]). In summarizing his and Rausch’s (1950 [42]) early research at Barrow, an apparent pattern was developing as 1946, 1949, and 1953 were brown lemming population highs. There was no mention of collared lemmings.

### 5.1. Barrow Lemming Cycles 1955–1974

The next and most significant period of lemming research was from 1955 to 1974 by Frank A. Pitelka (Pitelka 1957 [10,11], Pitelka 1973 [27], Batzli et al., 1980 [48], Pitelka and Batzli 1993 [28], Pitelka and Batzli 2007 [16], Pitelka and Batzli 2018 [17]). I termed this the Pitelka Years. Most of these data derive from 22 years of snap-trapping brown and collared lemmings during early, mid, and late summer, at sites within 5 km of the Naval Arctic Research Laboratory (NARL), at Barrow. Trapping was also conducted occasionally just beyond Barrow and in other areas of the North Slope. This study was originally led by Pitelka and later by Pitelka and George O. Batzli.

Shortly after Pitelka’s study began, a lemming population high was inferred in 1956. Pitelka then pooled information and data from 1946 to 1957 (Rausch 1950 [42], Thompson 1955 [34], Pitelka 1957 [10]) and concluded that lemming population highs occurred in 1946, 1949, 1953, and 1956. Although Pitelka’s figure (p. 79, 1957 [10]) was a generalized apparently hand-drawn undulating ‘wave’ graph, with little quantitative data, it was convincing. He concluded the lemming cycle was at 3–4-year intervals.

### 5.2. Pitelka 1973 [27]

Pitelka’s first major paper on Barrow lemmings covered 1955–1972 (Pitelka 1973 [27]). However, Pitelka also included data from Rausch (1950 [42]) and Thompson (1955 [34]). Thus, depending on the topic of discussion, lemming population highs from 1946, 1949, and 1953 were included in some discussions, but not others. In this paper, Pitelka (1973 [27]) separated the data from 1949 to 1965 and 1966 to 1972. He used his trap data from 1955 to 1972 to make his cyclic inferences, but would also include peak lemming years of 1946 and 1949 (Rausch 1950 [42]) to buttress his cycle theory. For example, from 1946 to 1965, Pitelka concluded six lemming population highs occurred (1946, 1949, 1953, 1956, 1960, 1965) with an average interval of 3.8 years. There were concerns regarding 1956, as spring trap results were not typical of a population high, and lemmings may have begun to decline before trapping began (Pitelka 1973 [27]). Pitelka (1973 [27]) provided an explanation to support a 1956 lemming population high even though the trap results were relatively low. He felt early snow melt exposed lemmings to avian predation. Thus, he inferred a population high from the presence of avian predators and damage to vegetation and concluded 1956 was similar to the highs of 1953, 1960, and 1965. In doing so, he buttressed the 3–4-year cyclic theory.

In the next analysis, from 1966 to 1972, questions arose in the breakdown of the cycle as characteristics of typical lemming build-up years were not observed, and no population highs occurred (Pitelka 1973 [27]). Pitelka believed the winter/spring of 1968–1969 was a “suppressed peak” and would have been consistent with the four-year cycle, but populations never reached their potential (Pitelka, p. 208, 1973 [27]). He went on to say weasel predation and reduced access to winter food due to snow conditions interrupted the 4-year cycle, even though trap results did not indicate a peak spring lemming population. Pitelka used this explanation to maintain the cycle’s regularity. In 1970, brown lemmings were scarce, and Pitelka believed unusual weather patterns, coupled with predation and food, among other factors, were responsible (Pitelka 1973 [27]). Similarly, in spring 1971, environmental conditions and evidence of lemmings indicated an upcoming lemming population low. However, it was an unexpected population high, comparable to other highs (Pitelka 1973 [27]). In this case, previous factors that influenced population highs did not agree with the 1971 surprise. Thus, Pitelka concluded that good spring vegetation, lack of lemming winter nests, and few juvenile brown lemmings indicate lemmings must have immigrated (Pitelka 1973 [27]). And, for the first time, collared lemmings experienced a significant population high, albeit much lower amplitude than brown lemmings. Thus, 1971 puzzled Pitelka because not all factors were in place as in previous highs. The 1971 high was followed by a lower population in 1972. The lemming “cycles” observed from 1949 to 1965 had apparently broken down from 1966 to 1972 (Pitelka 1973 [27]).

Overall, Pitelka (1957 [10], 1973 [27]) first used 1946, 1949 (Rausch 1950 [42]), and 1953 data (Thompson 1955 [34]) to neatly piece together the 3–4-year lemming cycles. As his research grew over the years, however, he omitted the 1946 verbal communication of a lemming population high (sometimes), then omitted the 1949 lemming high (sometimes). He probably dropped these because 1946 was hearsay, and 1949 had no trap data—a reasonable decision. He included the 1953 lemming high for a while (Thompson 1955 [34]) but eventually dropped that data (sometimes) and then used only results from his own trapping data. These were all reasonable decisions.

From a reviewer’s perspective, however, more confusion arises because others (Bee and Hall 1956 [45], Maher 1970 [63], MacLean 1974 [64]) suggested some population highs that were not considered by Pitelka. For example, Bee and Hall (1956 [45]) outlined the lemming high of 1949 (Rausch 1950 [42]), then the lemming low and build up to 1952 in the Barrow region (Barrow Village, Birnik). Indeed, by early June of 1952, Bee and Hall considered that the lemming population had reached another high based on their snap-trapping and observations. They estimated 50 lemmings per acre (125 ha) in the best habitats, versus 20 per acre (50 ha) in less quality habitat, and lemmings were more evenly distributed throughout the tundra than in other years. Although the population fluctuated a bit during the summer of 1952, it was high going into winter 1952/1953.

Following Pitelka’s (1957 [10], 1973 [27]) lead, Maher (1970 [63]) reported that lemming peaks were reached four times between 1949 and 1960 (1949, 1953, 1956, 1960). Maher (1970 [63]) also suggested a moderate population increase for 1952, because Pomarine Jaegers (*Stercorarius pomarinus*)—a near obligate brown lemming predator—bred in significant numbers in 1952, as well as the high lemming years of 1953, 1956, and 1960. Interestingly, this agrees with Thompson’s (1955 [10]) 200-fold lemming increase in 1952, followed by an additional 2-fold increase for 1953.

Also, Bee and Hall (p. 80, p. 86, 1956 [45]) noted a June increase in lemmings, as well as Pomarine Jaegers nests, and considered 1952 a population high which is not mentioned in any lemming population “cycle” summaries from Barrow. So, why was only 1953 considered a population high? What about 1952? Thus, perhaps back-to-back lemming population highs occurred in 1952 and 1953, with only slight variation in amplitude.

Even Batzli et al. (p. 338, Figure 10.2, 1980 [48], reproduced below) show question marks over 1956, 1963, and 1968 as population highs, and a population high for 1972—but not with a question mark. The question marks arise from the amplitude of lemmings at the time of trapping. There were other years with lower amplitude (1964, 1972) that could have been interpreted as a lemming population high; however, they were not considered or fit the pre-determined definition of a population high by Pitelka (p. 203, 1973 [27]). These data were expressed graphically in later papers that are discussed below. In particular, 1964 appeared to be a lemming population high, and was followed by a higher year in 1965, but 1964 was not considered. One could conclude that 1964 and 1965 were also back-to-back lemming highs, although the amplitude varied (Batzli et al., p. 338, Figure 10.2, 1980 [48]).

### 5.3. Batzli et al. (1980) [48]

**Figure 1:**
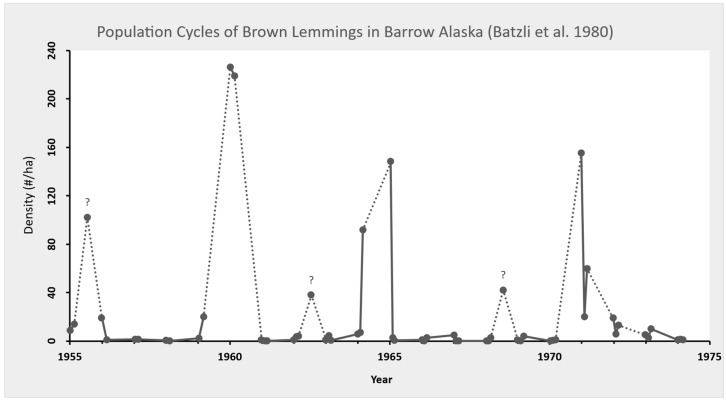
From Batzli and Pitelka (p. 338, Figure 10.2, 1980, [48]). The vertical (Y) axis represents the number of lemmings per hectare (ha). The three question marks (?) over the line-graph could have been lemming population peaks, but trap data did not align with the definition of a peak.

After the 1973 paper, a review of the herbivore-based trophic system on the coastal tundra was published (Batzli et al., 1980 [48]). In this manuscript, the authors concentrated mostly on lemmings and caribou (*Rangifer tarandus*). They included twenty years (1955–1974) of data for lemmings. Their review concentrated mostly on the nutrition and energetics of lemmings. However, they did reference in detail population dynamics and demography, and extrinsic and intrinsic factors influencing lemming populations (e.g., predation, physiology, genetics). In fact, they reiterated many of the results from Pitelka’s earlier works and concluded lemming peaks occurred every 3–6 years, with population lows lasting 1–3 years. They did not include 1956 as a lemming high because trap results did not indicate a high. In fact, they concluded spring numbers of lemmings in 1960, 1965, and 1971 were population highs, whereas spring numbers of lemmings in 1956, 1963, and 1968 declined before maximum densities could be assessed. They attributed these declines to avian and mammalian predation. Interestingly, the population high of 1960 lasted all summer. Their graph (p. 338, Figure 10.2, reproduced below as Figure 1) shows the trapping results and displays the highs of 1960, 1965, and 1971, and their uncertainty (shown with a question mark), if 1956, 1963, and 1968 were population highs, just of lower amplitude. In summing the lemming data from Barrow, between 1946 and 1974, Batzli et al. (p. 338, 1980 [48]) stated that lemming population fluctuations have “traditionally been called cycles, largely because of their great amplitude (3 or more orders of magnitude), even though all aspects of successive cycles are not alike.”

### 5.4. Batzli et al. (1981) [48]

In their 1981 paper on energetics of small mammals, the authors included the 1956 data (see Batzli et al., p. 338, Figure 10.2, 1980 [48]) with a question mark and suggested that observations other than trapping did indicate a lemming population high. In looking at Batlzi’s graphs, p. 383, 1981 [49], there were other years with lower amplitudes (1963, 1964, 1968, 1972) that could have been interpreted as a lemming population high; however, they were not considered or fit the pre-determined definition of a population high (Batzli p. 383, 1981 [49]). In particular, 1964 appeared to be a lemming population high, as the density estimates were almost identical to 1956, but 1964 was not considered a high because it did not meet their definition (Batzli p. 383, 1981 [49]). Then, in 1965, the lemming population was higher than in 1964 and considered a peak. Again, one could argue that 1964 and 1965 were back-to-back lemming highs, although the amplitude varied (Batzli et al., p. 338, Figure 10.2 1980 [48] and Batzli 1981, p. 383, [49]). Their graph leaves one wondering why 1963, 1968, and 1972 were not considered increases in lemming populations, just of lower amplitudes (Batzli p. 383, 1981 [49]). Alternatively, 1972 could have been considered a peak population, but lower than 1956 and 1964, and just a bit higher than 1963 and 1968 (Batzli p. 383, 1981 [49]). Thus, perhaps 1971 and 1972 were also back-to-back lemming population highs, just of lower amplitudes.

Finally, when the equation developed by Batzli (1981 [49]) was applied to Pitelka’s 1955–1973 (1973 was added in this paper) snap-trap data, Batzli concluded that brown lemming population peaks with >100 lemmings per ha occurred four times (1956, 1960, 1965, 1971) (Batzli 1981 [49]).

### 5.5. Pitelka and Batzli (1993) [28]

In their culminating research paper, together, Pitelka and Batzli (1993 [28]) reanalyzed the original data (1955–1972) and added data from 1973. For this analysis (1955–1973), Pitelka and Batzli (p. 218, 1993 [28]) defined the multi-annual cycle of Microtine rodents as “periodic episodes of high population density followed by very low population density (the extremes in density spanning more than a ten-fold difference).” In this paper, they applied a statistical method to their lemming trapping data that analyzes periodicity. This method was believed applicable for detecting cycles and requires at least four years of data. To determine if lemmings at Barrow were cyclic, Pitelka and Batzli (1993 [28]) applied the cyclic index developed by Henttonen et al. (1985 [58]) for studies of *Clethrionomys* voles in Europe. This technique yields a value where if s > 0.50, the population of interest is cyclic; if s < 0.50, the population is not cyclic (see Pitelka and Batzli 1993 [28]). However, Pitelka and Batzli (1993 [28]) also stated that the amplitude of cycles may be reliable in recognizing cycles in a shorter time frame (see Pitelka and Batzli 1993 [28]).

In this paper, Pitelka and Batzli (1993 [28]) first analyzed their capture data as an index of density, using number of captures per/100 trap nights. Because 1956 data did not meet the index definition of a peak population, they dropped the 1956 lemming population high and concluded only three population highs occurred between 1955 and 1973 (1960, 1965, 1971). Furthermore, their cyclic index, s = 0.79, supported the trap data, indicating a cyclic population for brown lemmings. They concluded brown lemming population highs occurred every 5–6 years. However, Pitelka and Batzli (1993 [28]) inherently believed 1956 was also a high year, but lemming populations declined so rapidly, trapping results and the cyclic index did not detect the high. They also suggested similar events for 1963 and 1969. Thus, in their summary, the authors deferred to their original trap data, field experiences, and qualitative explanations and considered all years, including the 1949 and 1953 highs (Rausch 1950 [42], Thompson 1955 [34]), the 1956 inferred high (Pitelka 1973), and 1963 and 1969 highs, and concluded population cycles for brown lemmings in the Barrow region occurred every 2–4 years (Pitelka and Batzli 1993 [28]), and not 5 to 6 years as stated above. Surprisingly, the cyclic index, s = 0.64 for collared lemmings, indicated they too were a cyclic population. Pitelka and Batzli (1993 [28]) concluded that reaching high densities is not necessary for a cyclic population; however, those species that do reach high densities are more likely to cycle.

Similarly, in reviewing Pitelka’s (1973 [27]) Barrow data from 1955 to 1972, Krebs (1993 [15]) graphed the number of lemmings (log scale) caught per trap line against year. Krebs (1993 [15]) concluded that 1956, 1960, 1965, and 1971 were lemming population highs—every 4–6 years, which agreed with much of Pitelka’s work. However, Krebs questioned if 1968 was also a peak population (Krebs p. 249, Figure 1, 1993 [15]). The Krebs (1993 [15]) paper was also published in the same proceedings as Pitelka and Batzli (1993 [28]).

Thus, depending on the analyst, and the years used, readers of these papers could conclude the lemming cycle at Barrow is paced at 3–4-year intervals if pre-Pitelka data and Pitelka 1955–1972 data are included (Pitelka 1973 [27]); 4–6-year intervals if only 1955–1972 Pitelka data are included (Pitelka 1973 [27]); 4–6 years if Krebs (1993 [15]) log scale reanalysis of Pitelka’s 1973 data are used; 5–6-year intervals if the cyclic index model is used on data between 1955 and 1973 (Pitelka and Batzli 1993 [28]). Finally, in their last research paper together, they also suggest the cycle could range from 2 to 4 years if data from 1946 to 1973 are used (Pitelka and Batzli 1993 [28]). Collectively, the above results indicate the brown lemming cycle could occur at intervals of 2–6 years at Barrow.

Interestingly, the cyclic index value for collared lemmings revealed the population is also cyclic (Pitelka and Batzli 1993 [28]). This is hard to believe, as except for 1971, few collared lemmings were caught during 19 years of trapping (Pitelka p. 203, Table 1, 1973 [27], Pitelka and Batzli p. 221, Figure 2, 1993 [28]). In fact, zero collared lemmings were caught during 7 of the first 11 years, but captured every year after 1965. Thus, do collared lemming populations cycle every 19 years at Barrow?

### 5.6. Pitelka and Batzli (2007) [16]

In 2007, Pitelka and Batzli (2007 [16]) published another paper briefly outlining a historical overview of their research on lemmings at Barrow. They suggested three factors that influenced the timing and amplitude of brown lemming cycles at Barrow: (1) vegetation; (2) predation; and (3) wet summers, freezing-rain winters, or winter thaws. In this paper, they first review lemming research at Barrow from 1946 to 1955. Next, they review lemming research from 1955 to 1974, which included all their peer-reviewed papers and their students’ theses and dissertations. They suggested the lemming population peaked every 3–5 years (p. 323) or 3–4 years (p. 328) during the early years of study.

They went on to address the importance of long-term study and used all the data available from about 1946 to 1974. They briefly reference the peak years and low years and try to explain the causes in relation to lemming cycles. These explanations are similar to previous discussions by them. Here, however, they outlined in more depth their thoughts on the Nutrient Recovery Hypotheses’ (NRH) role in lemming cycles. They also discussed the use of models in describing lemming cycles and concluded there were mixed results, but emphasized predation and the relationship between lemmings and forage. Basically, however, they concluded that multiple factors affect lemming cycles at Barrow. They recommend that future lemming studies must include winter aspects of their ecology. Interestingly, Pitelka’s reference (Pitelka and Batzli, p. 325, 2007, [16]) to Charles Elton, Victor Shelford, and David Lack suggests he was personally influenced by their outlooks on animal cycles.

### 5.7. Pitelka and Batzli (2018) [17]

Finally, in 2018, Pitelka and Batzli (2018 [17]) published another long paper dealing primarily with seasonal and cyclic changes in brown lemming demography, in relation to population fluctuations. They examined the body condition of 19, 428 necropsied lemmings they snap-trapped and hand-captured over 22 years at Barrow—an enormous number. They examined lemming carcasses for sex, body length, body mass, fat content, wounds, and reproduction. They used these data to infer and buttress their arguments related to lemming population cycles at Barrow. They concluded there were three seasonal periods within a year of lemming reproduction: (1) 2.5 months of intense summer breeding; (2) 6.5 months of moderate breeding during winter; and (3) 1.5 months during spring and autumn, with little or no breeding. They suggested brown lemming cycles at Barrow were divided into four annual phases: increase, peak, decline, and low.

For population structure (age), they assigned lemmings as juvenile, sub-adult, and adult, based on body length. Based on carcass data, they also provided data on the body mass of adult males and non-pregnant females. Pregnant females were used to provide litter size. Both sexes were used to assess the sex ratio.

Basically, they used the carcass data to support their arguments regarding four annual phases of lemming cycles at Barrow (increase, peak, decline, and low). They emphasize the Nutrient-Recovery Hypotheses—that higher quality forage is an important factor in driving lemming population cycles at Barrow. They suggest that foraging by lemmings in peak population years reduces the quantity and quality of food—which in turn takes time to recover—thus affecting lemming population cycles and the four annual phases.

They concluded that predation also has important effects on lemming populations. Indeed, predators such as Pomarine Jaegers, Snowy Owls, and least weasels (*Mustela nivalis*) can have significant effects on lemming population structure during summer and winter. They suggest that weather variables and changing snow and rain conditions can have impacts on lemming survival, but to a lesser extent. Finally, they conclude that social strife had little effect on the lemming population structure.

## 6. Lemming Life History Traits Associated with Cycles at Barrow

Clearly, brown lemmings at Barrow were one of the most thoroughly studied populations of *Lemmus* in the world. Here they experienced one of the highest amplitudes and density fluctuations of any North American lemming population. In addition, the incidental collared lemming research at Barrow offers important insights into their ecology as well.

Pitelka and colleagues’ research was monumental. However, lemmings did not always cooperate and do what Pitelka and others expected. So, Pitelka and others used deductive reasoning to explain unexpected events in a qualitative way. I believe this was a reasonable and legitimate means to discuss and assess one’s data but sometimes deductive reasoning and data are a fuzzy match. In fact, Pitelka and Batzli offered several different conclusions regarding lemming cycles at Barrow, depending on their analysis. However, other researchers at Barrow also offered slightly different conclusions than Pitelka and Batzli, or overlapping interpretations about lemming population highs, depending on their topic of interest. In general, there was a reluctance by Pitelka and Batzli to acknowledge that lemming populations may not cycle in a clear and predictable pattern, but qualitative explanations were presented to keep the lemming cycle theory alive. In fact, the annual phases of a cycle (increase, peak, decline, low) were not always sequential, even if the concept had merit. For example, sometimes peak populations could have been back-to-back, or low populations extended for several years, or even an unexpected peak that was supposed to be a low season. For more details on the above sections, see Pitelka (1973 [27]), Batzli et al. (1980 [48]), Batzli 1981 [49].

### 6.1. What Caused Lemming Population Highs at Barrow

At Barrow, much attention has focused on lemming declines, and few papers (Pitelka 1973 [27], Batzli et al., 1980 [48], Pitelka and Batzli 2018 [17]) discuss in detail the events leading to a lemming population high. In general, plentiful food, summer survival, vegetative cover, reduced summer predation, adequate snowpack, lack of subnivean predators, over-winter survival, large body mass, winter breeding, and favorable spring weather were thought to influence spring population highs at Barrow (Bee and Hall 1956 [45], Pitelka 1973 [27], MacLean et al., 1974 [64], Batzli et al., 1980 [48], Pitelka and Batzli 2007 [16], Pitelka and Batzli 2018 [17]). Yet, summer population highs were not always indicative of upcoming winter breeding, and winter breeding was not always indicative of spring and summer population highs (Bee and Hall 1956 [45], Batzli et al., 1980 [48]). There was a general trend for summer breeding to slow down or cease by September, or at the onset of freeze-up (Batzli et al., 1980 [48]). Winter breeding was thought to last from November to April, although a smaller percentage of females were pregnant—sometimes none—than in summer when most females were pregnant (MacLean et al., 1974 [64], Batzli et al., 1980 [48]). Additionally, adult female litter size was smaller in mid-winter (*n* = 3) than in summer (*n* = 8) (Osborn 1975, in Batzli et al., p. 341, 1980 [48]).

#### Comparisons with Relevant Literature

Unfortunately, there has been little research on the purported build-up phase of a lemming cycle, particularly what happens in winter (Chitty 1996 [4], Krebs p. 54, p. 239, p. 445, 2013 [5], Pitelka and Batzli 2018 [17], Erich et al. (2020 [18]), Gauthier et al. (2024 [19]). One of the first to report the increase phase of a collared lemming cycle correlated with increased snow depth was Shelford (1943 [9]). Later studies indicated winter breeding is uncommon except during the increase phase of population cycles (Krebs 1964 [12], Pitelka 1973 [27], Pitelka and Batzli 1993 [28], Chitty 1996 [4], Pitelka and Batzli 2018 [17], Gauthier et al. (2024 [19]) and this appears to explain the peak phase (see Chitty 1996 [4], Krebs 2013 [5], Pitelka and Batzli 2018 [17]), providing adequate snow covers exists (Shelford 1943 [9], Ims et al., 2011 [65], Gauthier et al., 2024 [19]).

### 6.2. Synchrony Among Population Highs and Lows at Barrow and the North Slope

Pitelka and colleagues also trapped small mammals at other villages on the North Slope of Alaska (Pitelka and Batzli 1993 [28]). These studies were conducted north of 70° 30′ and were intended to define species distribution and compare population cycles for interspecific and intraspecific synchrony (Pitelka and Batzli 1993 [28]). Over the long term, comparisons among sites revealed no regional synchrony in cycles, particularly during lemming population highs. For example, in 1957, the population high of brown lemmings at Wainwright was not synchronous with Barrow, 140 km northeast (Pitelka and Batzli 1993 [28]). Occasionally, however, widely separated populations did reach population highs synchronously (Pitelka and Batzli 1993 [28]). In 1960, brown lemming populations were high at Barrow, synchronous with Pitt Point, 150 km southeast, but not Wainwright, 140 km southwest (Pitelka and Batzli 1993 [28]). In 1963, lemming populations at Wainwright and Inaru had a moderate synchronous peak, although separated by 125 km (Pitelka and Batzli 1993 [28]). Also of interest was a moderate but consistent lemming population high from 1960 to 1963, at Wainwright (Pitelka and Batzli 1993 [28]). Overall, Pitelka and Batzli (1993 [28]) found little support for synchrony among small mammal populations over a wide geographic range on the North Slope of Alaska. These results agree with Bee and Hall (p. 92, 1956 [45]), who also suggested little synchrony over wide geographic ranges on the North Slope. They suggested that habitat quality and competition with other Microtine rodents influence the magnitude of populations.

When looking at Pitelka’s trap index data between 1955 and 1973, mixed results occurred regarding population fluctuation synchrony among the two species of lemming at Barrow. Although collared lemmings were never abundant, it remains difficult to assess if brown and collared lemming populations were in synchrony. Few collared lemmings (*n* = 26) were trapped during the first 15 years of study (1955–1969), and zero were caught in some years (Pitelka p. 203, Table 1, 1973 [27]). In 1970, 1971, and 1972, 33, 176, and 15 collared lemmings were caught, respectively. In 1970, more collared lemmings (*n* = 33) were caught than brown lemmings (*n* = 6). In 1971, collared lemming populations reached a population high synchronously with a brown lemming population high. In all other years, collared lemming populations did not fluctuate synchronously, or they were not caught in traps, or their amplitude was so low that comparisons may not be meaningful. Thus, during 19 years of study at Barrow, only one year (1971) did the two species of lemmings reach a population high synchronously (Pitelka p. 203, Table 1, 1973 [27], and Pitelka and Batzli p. 221, Figure 2, 1993 [28]). And, except on rare occasions, data from other areas of Alaska did not indicate synchrony among brown and collared lemming population highs and lows over a wide geographic area (Bee and Hall 1956 [45], Pitelka and Batzli 1993 [28]). Interestingly, neither Rausch (1950 [42]) nor Thompson (1955 [34,44]) mentions any population high of collared lemmings from their studies at Barrow.

#### Comparisons with Relevant Literature

Historically, researchers believed lemming population cycles (intra-specific and inter-specific) were synchronous over wide geographic areas (Elton 1942 [1], Shelford 1945 [7], Krebs 1964 [12]). Even region-wide irruption migrations of Snowy Owls were thought to coincide with a 4-year region-wide lemming cycle. Kerlinger et al. (1985 [66]) results, however, did not support this cyclic association. Unfortunately, there is usually no accompanying data on lemming numbers at a region-wide scale. Chitty (1996 [4]) did not believe widespread population synchrony in lemmings occurred. And Gruyer et al. (2008 [30]) had equivocal results when looking at the synchrony of *Lemmus* and *Dicrostonyx* cycles over 13 years on Bylot Island, Canada. Sometimes, however, lemming population highs do appear to synchronize over large geographic areas in the Canadian Arctic (Krebs 1964 [12], Krebs et al., 2002 [57], Krebs et al., 2011 [31], Krebs 2013 [5], Krebs 2024 [20]) but this is not always consistent (Krebs et al., 2011 [32], Krebs 2013 [5], Erich et al., 2020 [18], Gauthier et al., 2024 [19], and this review). A more difficult question is what factors drive synchrony on a local and larger geographic scale, such as dispersal and weather effects (Krebs, p. 212, 2013 [5]). On the other hand, the continent-wide Snowy Owl irruption migration of 2010/2011 could have been the result of a rare widespread synchronous lemming population high. Interestingly, the same synchrony and cycle theory was applied to North American lynx and snowshoe hare population fluctuations (Chitty 1996 [4]). But upon closer examination, even the purported 10-year lynx and snowshoe hare cycle shows variation of 7–12 years, depending on location (Chitty 1996 [4]) or 8–10 years (Krebs 2011 [56]).

### 6.3. Migration, Immigration, and Emigration at Barrow

Although immigration, emigration, and mass movements have been reported at Barrow (Brower 1942 [41], Thompson 1955 [34], Pitelka 1973 [27]), it remains unknown what these observations really mean. Indeed, it is difficult to believe the magnitude of these observations due to the manner in which they were described. For example, at the end of May 1888, Charles D. Brower wrote that the lemmings came from the southeast, “first in scattered bands, then solid masses”. The tundra was “black” with lemmings. “You couldn’t put your foot down without stepping on a lemming.” They moved seaward on a 10-mile front and took four days to pass Barrow. They marched onto the ice and eventually “leaping into the water” where they drowned. While riding their boats several miles offshore, Brower also wrote “our bow swished for hours through great masses of drowned lemmings” and “millions must have drowned” (see Brower, p. 123, 1942 [41]).

Even researchers could not control embellishments. At Barrow in June 1953, a mass movement of lemmings attracted much attention, and it was “apparent there was mass unrest in the population.” (Thompson 1955 [34]). Many lemmings were reported on the gravel roads, in construction workers’ camps, and they moved from the shore to the ocean. The lemmings appeared in “haphazard dispersal or shuffle”. In fact, lemmings were reported from shoreline 10 miles onto the ice, and the lemming “front” extended 30 miles southwest, and 30 miles southeast, from the Barrow coast (Thompson 1955 [34]).

This particular event was called an emigration by Thompson (1955 [34]). He believed a small portion of the population moved seaward from the tundra to relieve overpopulation pressure. Thompson believed changes in food and cover were driving this phenomenon. Necropsies of captured “emigrant” and resident lemmings suggested emigrants were physically inferior to residents living on the surrounding tundra (Thompson 1955 [44]). Interestingly, there was a male-biased sex ratio among those emigrants. Bee and Hall (p. 88, 1956 [45]), however, felt there was no true migration and that local lemming movements seen in spring were shifts from winter range to summer range.

The only other mention of lemming mass movements from Barrow was reported by Pitelka (1973 [27]). He could not explain the surprise population high of 1971, as it did not fit the previous patterns. He suggested there must have been immigration from elsewhere on the tundra (Pitelka 1973 [27]). However, MacLean et al. (1974 [64]) were not convinced that the 1971 lemming migration took place. Rather, based on the number of lemming winter nests, MacLean et al. (1974 [64]) believed brown lemmings bred during the winter 1970/1971, causing the population high.

#### Comparisons with Relevant Literature

Mass movements of lemmings have been reported for over a century from various areas of the world (Elton 1942 [1], Chitty 1996 [4]). For example, Alaskan natives recollect mass movements of lemmings about once in ten years (Thompson 1955 [44]). On the Yukon River, Alaska, a floating mass of dead brown lemmings was observed in 1918 (Stuck 1920 [67]), and in Canada, an emigration of one lemming per square yard took 10 days to pass (Gavin 1945 [68]).

Krebs (1993 [15]) was skeptical regarding the 1971 Barrow migration and challenged Pitelka (1973 [27], Pitelka and Batzli 1993 [28]) explanations. Overall, Chitty (1996 [4]) too was unconvinced of long-distance lemming migrations, but believed short-distance movements were possible. Also see Krebs (2013 [5]) for discussion on immigration and emigration.

True mass movements of lemmings, however, are apparently associated with the European/Norwegian lemming (*Lemmus lemmus*) in the Scandinavian countries. This behavior is a seasonal elevation shift and appears to be characteristic of dense populations, and apparently a directional movement (Pleske 1884 [69], Collett 1895 [6], Elton 1942 [1], Kalela 1949 [70], Wildhagen 1952 [71], Kalela and Koponen 1971 [72], Batzli 1981 [49], Krebs 1993 [15], Chitty 1996 [4]). See also Stenseth and Ims (1993 [3]).

### 6.4. Density Estimates and X-Fold Increases and Decreases at Barrow

Estimating lemming density per unit area is highly desirable for population studies and ecological correlates. Several methods were described from Barrow, and results vary widely (see Table 1). For example, Bee and Hall (1956 [45]) believed 1952 was a lemming population high and estimated spring numbers at 50 ac (125 ha) in optimum areas and 20 ac (50 ha) in other areas. And, during the lemming population high of 1953, Thompson (1955 [34]) noted that lemmings were wandering onto the gravel streets of the Naval Arctic Research Laboratory. He established an 80-acre (32 ha) plot at a camp for sampling density. Based on catches, he estimated 1 lemming/per acre (0.50 ha) over a five-day period, and the average daily catch was 1 lemming per 5 acres (2 ha). He then sampled the adjacent tundra for a comparison and reported 42 lemmings per acre (105 ha) (Thompson 1955 [34]).

In early June 1960, the lemming population was estimated at 125 lemmings per ac (312 ha); however, by late August, it was estimated at 50 ac (125 ha). In June 1961, estimates were 0.5 ac (~1 ha); in June 1962, about 1–10 ac (~2/3–25 ha); and in June 1963, 50 ac (125 ha), which declined to 1–5 ac (~2/3–12/13 ha) by August. Elsewhere, scattered populations of 5–10 ac (12/13–25 ha) lasted all summer. There was no data for 1964. Finally, in June 1965, the lemming population was estimated at 50–75 ac (125–185 ha). See Mullen (p. 4, 1968 [73]). Also see Mullen and Pitelka (1972 [74]). Over the long term, between 1950 and 1965, Pitelka (p. 205, Figure 2, 1973 [27]) estimated 50–100 lemmings per 0.4 ha in “cyclic” peak years, and 1 lemming per 4.0 ha in low years (see Table 1).

Similarly, from 1952 to 1960, Maher (1970 [63]) estimated spring/June numbers of lemming in the high population years of 1953 at 70–80 ac (175–200 ha); 1956 at 40–50 ac (100–125 ha); and 1960, at 70–80 ac (175–200 ha), respectively. In low years, relative spring density was ~ 5 lemmings per acre (12/13 ha). Finally, during the period 1955–1974, brown lemming densities were estimated at 0.02 ha^−1^ to 225^−1^ ha, and collared lemmings 0.1 ha to 27^−1^ ha (see Batzli et al., 1980 [48]) (see Table 1).

Based on live trapping grids just before snap-trapping, Batzli (1981 [49]) developed a lemming density equation related to snap-trapping results. This equation was STI = 0.22 D^0.70^, where STI = snap-trapping index, and D = density. When this equation was used to calculate densities, the average error was 1.8 ha^−1^. Calibrations were not done for densities > 25 ha^−1^. Extrapolation to higher densities likely reduces accuracy (Batzli 1981 [49]). When using lemming nests as an indicator of density, MacLean et al. (1974 [64]) estimated 0 per ha in 1969/1970, 42 ha^−1^ in 1970/1971, 27.5^−1^ ha in 1971/1972, and 14.4 ha^−1^ in 1972/1973.

#### Comparisons with Relevant Literature

Other researchers also estimated percent-fold increases in small mammals from population lows to population highs. For example, Krebs (1964 [12]) estimated the amplitude change in one brown lemming cycle to be 50-fold, and one collared lemming cycle to be 25-fold. Krebs (1993 [15]) believed these estimates were reasonable and that estimates of 200-fold increases were possible when employing a simple life table. In reanalyzing Pitelka’s (1973) snap-trapping data from Barrow, Krebs (p. 250, 1993 [15]) estimated increases in June trap results following peak lemming years to be 113-fold (1956), 64-fold (1960), 29-fold (1965), and 47-fold (1971). However, Krebs (p. 250, 1993 [15]) also believed reports of 500:1 and 1000:1 change in amplitudes from Barrow (Pitelka p. 205, 1973 [27]) and (Batzli et al., p. 338, 1980 [48]) were too high (Krebs p. 250, 1993 [15]).

The density estimates per unit of area from Barrow varied substantially depending on the technique used, such as observation, snap-trapping, live trapping grids, or winter nest counts (see Density Estimates and X-fold increases). Batzli’s (1981 [49]) lemming density equation, based on live trapping results and then applied to snap-trapping results, seemed to be a reasonable approach. Krebs et al. (2002 [57]), however, believed reliable density estimates from snap-trapping were difficult if not impossible to achieve. Nonetheless, Krebs et al., 2002 [57] defined a peak lemming cycle in the High Arctic as 10 lemmings/ha or 3/per/100-trap-nights, or when the density of a species is higher in one year than the preceding or following year. More recently, however, Krebs developed a regression equation that provides a useful density estimator from snap-trapping results (C. Krebs, pers. comm., and Krebs et al., 2011 [31]).

### 6.5. Causes of a Lemming Population Decline at Barrow

Numerous hypotheses exist to explain the declines of lemming populations at Barrow. For example, Rausch (1950 [42]) and Thompson (1955 [34]) thought predation and lack of food contributed to the decline, while Thompson (1955 [34]) also believed least weasels could have been the primary cause. He ruled out parasites or disease as playing a major role. Bee and Hall (1956 [45]) mentioned several factors that could influence a “crash” and thought food supply was the major factor. In general, Pitelka (1957 [10,11]) attributed predation, lack of food, cover, and weather as contributing factors to most population declines. Nutrition, predation, and endocrine factors have also been implicated (Schultz 1964 [75]). Weasel predation was also believed to be an important factor impacting lemming population cycles, but winter weather was also considered (MacLean et al., 1974 [64]). Physiological and demographic effects were also suggested to influence population declines (Andrews et al., 1975 [76]). Lastly, a Nutrient-Recovery Hypothesis (NRH) was suggested by Pitelka (1964 [77]), defined by Schultz (1964 [75]), and reviewed and modified by (Batzli et al., 1980 [48]). In fact, Pitelka and Batzli (2007 [16]) concluded the NRH alone did not explain the lemming cycles at Barrow; however, they concluded the NRH “plays the most significant role in seasonal changes in brown lemming populations”. Predation plays a smaller role, and social strife had little effect (Pitelka and Batzli, p. 225, 2018 [17]).

From 1956 to 1960, Maher (1970 [63]) researched Pomarine Jaegers at Barrow. Maher concluded that lemmings were the driving force behind Pomarine Jaeger nesting density and productivity. Pomarine Jaeger nest density was highest during high lemming years, and brown lemmings made up about 80% of the Jaegers’ diet. Maher constructed a hypothetical Jaeger–lemming predation effect model using various lemming density estimates. He concluded avian predators (i.e., jaegers, owls, and gulls) could “truncate” a population high of lemmings, but not reduce populations to a low phase of the cycle. He suggested mammalian predators, especially least weasels, reduced lemming populations until the weasels declined in number, and the lemming populations recovered.

From 1969 to 1973, MacLean et al. (1974 [64]) studied predator–prey relations between short-tailed weasel (*Mustela erminae*), least weasel, and lemmings. MacLean’s objective was to determine if weasel predation affected lemming population cycles. In MacLean’s study, most weasel predation on brown lemmings was inferred from the use of lemming nests by weasels. They concluded that least weasels were closely linked to lemming cycles, whereas short-tailed weasels were uncommon and did not significantly prey on lemmings (MacLean et al., 1974 [64]). In other studies, least weasels were believed to be more common in high lemming years, and perhaps rare in low lemming years (Thompson 1955 [34], Pitelka et al., 1955 [78]).

Approximately 50 years ago, researchers at Barrow cautioned that climate change could affect snow quality, which is important for winter breeding of lemmings. Thus, any climate change-related effects on snow quantity and quality could affect lemming populations (Fuller 1967 [79], MacLean et al., 1974 [64], Batzli 1981 [49]). Currently, no analysis has been conducted in this region.

#### Comparison with Relevant Literature

Each lemming population high and low has its own demographic signature, and numerous factors—abiotic and biotic—are believed to influence the decline. Most researchers conclude that predation by avian and mammalian predators has a significant impact (Shelford 1943 [9], Rausch 1950 [42], Pitelka 1957 [10,11], Maher 1970 [63], MacLean et al., 1974 [64], Krebs 2013 [5], Pitelka and Batzli 2018 [17]. However, food, physiology, and population demographics are also suggested (Andrews et al., 1975 [76], Schultz 1964 [75], Batzli 1980 [49], Krebs 2013 [5], Pitelka and Batzli 2018 [17]). Unfortunately, individual traits such as sex, mass, and relative age of lemmings killed by predators are usually unknown. This would be important in determining effects and effects on predation on the lemming population structure. In a review of factors influencing lemming and other small mammal population dynamics, Chitty (1996 [4]) added adrenal function, congenital function, disease, epidemics, fighting, genetics, malnutrition, maternal stress, natural selection, parasites, spleen function, sex-specific behavior, starvation, sun-spot cycles, and weather. Apparently, overpopulation can lead to many of these traits, which in turn could affect lemming populations. Many of these ideas have been tested, and none seem to fully explain the population changes (see Krebs 2013 [5]).

In one of the first reports implicating snow conditions, Shelford (1943 [9]) recorded a decline in collared lemmings associated with the absence or reduced amounts of snow cover. More recently, fluctuating snow conditions related to climate change are believed to have affected collared lemming, European/Norwegian lemming, and gray-sided vole (*Myodes rufocanus*) population cycles (Kausrud et al., 2008 [80], Gilg et al., 2009 [81], Karell et al., 2009 [82], Ims et al., 2011 [65], see review Erich et al., 2020 [18]). In response to these declining lemmings, declines in Arctic fox and Snowy Owls numbers in Norway, and declines and disappearance of Arctic fox, Long-tailed Jaeger (*S. longicaudus*, stoat/short-tailed weasel, and Snowy Owls in Greenland, are attributed to changes and declines in lemming populations due to climate change (Kausrud et al., 2008 [80], Gilg et al., 2009 [81], Schmidt et al., 2012 [83], Erich et al., 2020 [18]). And Ims et al. (2011 [65]) recently stressed the importance of snow cover for winter breeding in lemmings. He believed winter breeding in response to 8–10 months living under snow evolved to avoid summer predators. Thus, changes to snow conditions could influence spring population highs at northern latitudes and alter lemming life history. On the other hand, Erich et al. (2020 [18]) found no evidence that climate change negatively affected lemming populations on a global scale. And Gauthier et al. (2024 [19]) reported that changing snow conditions due to climate change had no effects on lemming cycles.

## 7. Is There an Association Between Lemmings and Other Wildlife Species at Barrow?

### 7.1. Is There an Association Between Lemmings and Other Wildlife Species at Barrow?

Iñupiat Eskimos, early explorers, and early wildlife researchers at Barrow clearly recognized both qualitatively and quantitatively that when lemming populations were high, other species of birds and mammals seemed to correspond (Brower 1942 [41], Bailey 1948 [84], Pitelka et al., 1955 [78,85]). In fact, the number of Pomarine Jaeger “pairs” increased with increasing lemming density (Maher 1970 [63]). Glaucous Gulls (*Larus hyperboreus*) also fed on lemmings and were relatively absent from the tundra during lemming lows (Rausch 1950 [42]), but nested in higher numbers when lemmings were abundant (Pitelka et al., 1955 [78], Pitelka 1957 [10]). And to a lesser degree, Parasitic Jaeger and Long-tailed Jaeger (Pitelka et al., 1955 [78]) increased with increasing lemming density. Common Ravens (*Corvus corax*) also preyed on lemmings when the ravens expanded their range to Barrow (Pitelka et al., 1955 [78]). Snowy Owls were also dependent on lemmings for breeding (Pitelka et al., 1955 [85]), as were Short-eared Owls (*Asio flammeus*) when they occasionally occurred (Pitelka et al., 1955 [78]).

Mammals, too, responded at Barrow. Winter numbers of Arctic fox appear to correlate with high densities of brown lemmings (Mullen and Pitelka 1972 [74]). The number of Arctic fox breeding dens and production was also linked to lemming populations (Batzli 1981 [49]). Least weasels were also more abundant when lemming populations were high (Maher 1970 [63], MacLean et al., 1974 [64]), and perhaps short-tailed weasel but that was not entirely clear (MacLean et al., 1974 [64]).

In the mid-1990s, field researcher Michele Johnson-Deering (MJD) made observations and suggested a relationship between Steller’s Eiders (*Polysticta stelleri*), Pomarine Jaegers, Snowy Owls, and lemming populations (MJD pers. comm.). Also from Barrow, Quakenbush et al. (2002 [86]) noted that previous shorebird researchers, and data gathered by MJD and others from 1992 to 1999, suggested Steller’s Eider nesting numbers were correlated with lemming population fluctuations. Quakenbush et al. (2004 [87]) did not study or sample lemmings or Snowy Owls; rather, they included unpublished data provided by the Owl Research Institute’s (ORI) lemming and Snowy Owl study (D. Holt unpubl. data). These ORI data were also used to buttress Quakenbush et al. (2004 [87]) suggestion of a brown lemming—Snowy Owl—Steller’s Eider association.

I am in complete agreement with MJD, and Quakenbush et al. (2002 [86]) and Quakenbush et al., 2004 [87]). After 35 years (1991–2025) of studying Snowy Owls and lemmings, my data clearly show Snowy Owls are a near-obligate lemming predator for breeding at Barrow, and elsewhere (Holt et al. (2024 [51]) and Holt et al. (2025 [88]). Furthermore, over the past 35 years, I have walked thousands of miles on the tundra researching lemmings and Snowy Owls during high and low population years of both species. My observations lead me to suggest that other species are influenced by population fluctuations in lemmings. These include White-fronted Goose (*Anser albifrons*), Brant (*Branta bernicla*), Tundra Swan (*Cygnus columbianus*), and King Eider (*Somarteria spectabilis*) which all nest in higher numbers and appear to have higher nesting success when lemmings were abundant. Peripheral breeding species whose distribution is primarily Russian, such as the threatened Spectacled Eider (*S. fischeri*) and the threatened Steller’s Eider, nest at Barrow in small numbers, but appear to have better reproductive success when lemming populations are high. Willow Ptarmigan (*Lagopus lagopus*), Sandhill Crane (*Grus canadensis*), Glaucous Gull, and numerous shorebirds and passerine species appear more abundant and/or have higher breeding output when lemming populations are high. Finally, at Barrow, there was even an indication that insects (Arthropods) may be more abundant when lemmings are abundant (Weber 1950 [62], MacLean and Pitelka (1971 [89]).

#### Comparison with Relevant Literature

There is convincing qualitative and quantitative evidence of an association between lemmings and other ground-nesting birds, particularly waterfowl, grouse, and shorebirds in Canada, Europe, Greenland, Russia, and Scandinavia. These include Brent Geese (Summers 1986 [90], Dhondt 1987 [91], Nolet et al., 2013 [92]), White-fronted Geese, Bean Geese (*A. fabalis*) (van Impe 1996 [93]), King Eider (Sittler et al., 2000 [94]), and Snow Geese (Tremblay et al., 1997 [95], Bêty et al., 2001 [96]). Similar results exist for grouse such as willow ptarmigan/red grouse in North America and Europe (Steen et al., 1988 [97], Moss and Watson 2001 [98]). In Russia, several shorebird species had better nesting success when “small mammal” populations were abundant (Summers and Underhill 1987 [99], Underhill et al., 1993 [100], Summers et al., 1998 [101]). Additionally, in Russia, reproductive success for geese, ptarmigan, other birds, and rabbits (*Lepus* spp.) was greater in high lemming years (Sdobnikov 1959 [102]). In Norway, ground-nesting birds and small rodents had population fluctuations in synchrony (Kausrud et al., 2008 [80]). In Greenland, Snowy Owls, Long-tailed Skuas, Arctic fox, and stoat were more abundant in high Lemming years (Gilg et al., 2009 [81]). Interestingly, in Canada, Dickson (1992 [103]) reported a relationship between voles (*Microtus*) population peaks and Red-throated Loon (*Gavia stellata*) hatching success over five years. In fact, all the above species appear to have higher nesting success in years of high lemming abundance.

## 8. Are Lemmings an Indicator Species for Birds and Mammals at Barrow?

### 8.1. Are Lemmings an Indicator of Arctic Ecosystem Health at Barrow

Lemmings influence the tundra ecology in the Barrow region perhaps more than any other species of birds or mammals. As the primary herbivore, cutting and consumption of Arctic vegetation affects all other species of ground dwellers. Cutting and consumption reduce ground cover for lemmings and ground-nesting birds and perhaps leave them more vulnerable to predation.

In times of low lemming populations, other current researchers have noted that avian and mammalian predators switch from primarily eating lemmings to eating eggs, chicks, and adult ground nesting birds (R. Lanctot and B. Kempenaers for shorebirds, and N. Graff for waterfowl; pers. comm.). In times when lemmings are abundant, observations suggest lemmings become the primary prey for breeding lemming dependent predators such as Snowy Owls Pomarine Jaegers, short-tailed weasel, least weasel, and Arctic fox (Pitelka et al., 1955 [78], Maher 1970 [63], MacLean et al., 1974 [64], Quakenbush et al., 2004 [87], Holt et al., 2025 [52]). Consequently, relaxed predation pressure on ground-nesting birds is believed to occur. In turn, their reproductive output increases. Lemmings also provide the highest animal biomass for predators, leading to higher reproductive success for them as well.

It is common knowledge at Barrow that early season (May/June) detections of large numbers of obligate lemming predators such as Arctic fox, Snowy Owls, and Pomarine Jaegers indicate an abundance of lemmings. And high populations of lemmings during spring snow melt can be an indicator of an upcoming productive breeding year for many species of birds and mammals, provided lemming populations remain high through spring and summer. The reasons are not entirely clear, but qualitative explanations are provided. During lemming population highs, there are many lemmings, and they are large and appear easy to catch for several species of avian and mammalian predators. Thus, predation pressure on ground-nesting birds is relaxed, leading to high breeding output.

#### Comparisons with Relevant Literature

Animals have always been used as barometers of environmental change. Many species have been labeled as bellwether, bio-indicators, capstone, flagship, focal, indicator, keystone, proxy, surrogate, trust, umbrella, and other definitions. The terms are often split into definable categories, yet conceptually they remain basically the same. For example, (Mills 2007 [104]) outlined four terms that define types of focal species commonly used in conservation biology: (1) flagship, (2) umbrella, (3) indicator, and (4) keystone. These categories and their definitions are confusing, interchangeable, and have mixed and overlapping meanings. We need a simple word to describe the basic concept. I personally like indicator species as a wide-ranging title that carries all definitions stated above. This term has been around for many years, and the word is understood by most people.

Lemmings may be the best indicator of an upcoming regionally productive breeding season and the overall health of the Arctic as the climate changes. However, lemmings are difficult to observe and have a rodent stigma. Thus, alternatively, I propose using Snowy Owls as the poster bird for Arctic conservation and an indicator of the general health of the terrestrial ecosystem. Snowy Owls are easy to observe, have high public appeal, high market value, and are obligate lemming predators throughout their worldwide distribution (Holt et al., 2024 [51], Holt et al., 2025 [52]). The fact is that certain animals have influencing effects on people. These animals render admiration, concern, and sympathy for their well-being for a variety of reasons. Indeed, using charismatic species such as some predators for landscape conservation and indicators of healthy environments has significant potential (Sergio et al., 2006 [105]).

## 9. Future Research Suggestions for Barrow

### 9.1. Long-Term Studies

Pitelka and Batzli conducted long-term research with enormous samples and clearly expert performance. Yet in this changing climate, we need a commitment to more long-term lemming studies at Barrow. These studies must be conducted by the same seasoned field researchers for 20 or more years. Unfortunately, the typical graduate study duration of 1–3 or 4 seasons cannot meet the scientific rigor needed for expert performance. In fact, the limits of graduate student research on small mammals were pointed out by Taitt and Krebs (pp. 572–575, 1985 [14]) in their review of *Microtus* cycle studies and reiterated many times by Krebs (2013 [5]). Also see Holt (2022 [106]) for a commentary on expertise and expert performance.

Nonetheless, small mammal populations have been estimated using methods such as casual observations, casual walking counts within a fixed area, casual walking counts along fixed transects, indices of abundance (e.g., ranks), standardized snap-trapping, and standardized grid sampling. Snap-trapping has been the most widely used method throughout the world and represents some of the best examples of long-term population fluctuations in lemmings and voles. Indeed, Krebs (Chapter one, 2013 [5]) graphed four long-term small mammal monitoring projects using indices of abundance (e.g., ranks), and snap-trapping programs that represented 31, 40, 41, and 61 years of monitoring, respectively. These data clearly show patterns of population highs and lows, both seasonal and annual, with peak populations generally occurring every 3–5 years. On the other hand, standardized live trapping grids using mark-recapture methods are considered by Krebs (2013 [5]), Elrich et al. (2020 [18]), and Gauthier et al. (2024 [19]) as the best method for recording density and population structure. See Gauthier et al. (2024 [19]) for long-term grid sampling from Canada. Unfortunately, grid sampling is labor-intensive, and few people want to dedicate a career to long-term research covering decades.

Krebs (1993 [15], 2013 [5]) suggested experiments be conducted to tease apart factors influencing small mammal population fluctuations. Indeed, some field experiments provide convincing results as to mechanisms influencing population fluctuations in small mammals. However, even experiments can be poorly designed and misleading, leaving us with confusing results (see review in Krebs (2013 [5]). Nonetheless, experiments must also be long-term, have large sample sizes, be multifactorial, conducted by seasoned field researchers, and be replicated numerous times by the same and independent researchers.

Modern mathematical analysis and advanced computer models can help in teasing apart long-term data sets. For example, Krebs (p. 11, p. 16, 2013 [5]) suggested time series analysis as a favorite method to examine rodent population fluctuations, but there must be 25 or more years of data. Unfortunately, few studies provide such data. Furthermore, Krebs (Chapter 13, 2013 [5]) reviewed the strengths and weaknesses of models and concluded that they are valuable in assessing population fluctuation in rodents, but data from natural populations are needed to test models against reality, and continuous feedback is needed to strengthen results. Ehrich et al. (2020 [18]) and Gauthier et al. (2024 [19]) used time series and spectral analysis to assess lemming population fluctuations in relation to trends and winter climate, respectively. Their results indicate stable lemming populations worldwide and further suggest that climate change has not affected lemming populations.

#### 9.1.1. Winter Studies

In 1956, Bee and Hall (1956 [45]), in reference to Barrow, stated that to understand lemming cycles, we need to uncover what biological factors take place beneath the snow during winter. This was reiterated by MacLean et al. (1974 [64]) for Barrow. Thirty-seven years after Bee and Hall’s statement, Krebs (1993 [15]) echoed the need for winter research on lemmings throughout their range. And Pitelka and Batzli (2007 [16]) argued again for winter research. Currently, 68 years later, we have no satisfactory research answer to that question for Barrow. Generally speaking, winter population information is largely deduced from counting winter nests (Gilg et al., 2009 [81], Fauteux et al., 2015 [33], Pitelka and Batzli 2018 [17], D. Holt, unpubl. data), and mechanisms influencing winter lemming population ecology remain largely unknown for Barrow. Nonetheless, efforts are being made to monitor winter lemming activity in other areas (see Soininen et al., 2015 [35]). However, given the difficulty in assessing this aspect of lemming ecology, reliable methods must be worked out over time so as not to be another short-term study with ambiguous results. These studies must also be long-term, even if challenging. If winter research proves too difficult and untrustworthy, then perhaps winter walking counts within a fixed area, grid, transects, observation of tracks, and presence of lemming predators could provide a relative index of abundance. The fact is, at Barrow, brown lemmings are seen with some regularity in town during winter, running across streets and from under house to house. Perhaps they surface on the tundra more than we think for reasons we do not know. In temperate latitudes, voles, also known to be subnivean, are regularly seen on the snow surface during winter. So, how else would Snowy Owls and other lemming and vole specialist predators survive winter conditions unless some of these small mammals surfaced?

In fact, some small mammal researchers have noted that “snow chimneys”—tunnels leading from the subnivean winter environment to outside ambient air—have been dug by lemmings and voles. This has led to the notion that CO_2_ build-up from animal and plant respiration builds enough gases that could become toxic. Clearly, data indicates lemmings and voles dig surfacing tunnels and venture onto the snowpack. The question is why? Does the gas really build up, or is it easier for these small mammals to wander on the snow surface than to move through the subnivean space? In some studies, CO_2_ build-up is negligible, while in other studies, it can be high. Whether this is the reason lemmings and voles, surface is unclear. Nonetheless, the behavior appears to occur with some regularity, and even tunnels are cleared after fresh fallen snow. Regardless of the driving forces behind this behavior, many small mammals do surface from under the snowy pack. See Batzli et al. (p. 373, 1980 [48]) and Marchland (pp. 178–184, 1996 [107]) for brief summaries of what the data imply. Nonetheless, snow chimneys or surface tunnels are thought to be more common during lemming population highs. If true, can this behavior influence Snowy Owl and other lemming predators’ decision to stay in an area or move on?

Also related to winter lemming life history, Bee and Hall (1956 [45]) provided a brown lemming body mass and pelage development growth chart (p. 100) based on 270 males and 270 females. During our >30 years of lemming trapping at Barrow, we trap small lemmings just emerging from snow melt in late June. It is conceivable to examine spring lemming body masses and pelage color with Bee and Hall’s growth chart and infer if lemmings bred under the snow during the previous winter. Indeed, perhaps lemmings breed under the snow more often than current data indicate, as our trap results suggest.

#### 9.1.2. Human-Influenced Habitat

At Barrow, there is a need for studies to be distanced from man-made structures and human habitation. For example, many recent studies on birds and mammals at Barrow are conducted adjacent to or within building complexes, field stations, fragmented habitats, paralleling road systems, or along man-made walkways and snow fences. These sites may not represent a natural ecosystem. The fact is, the tundra adjacent to the city of Barrow is littered with an enormous amount of old and new man-made structures and old equipment. These items have been discarded by local residents, former military presence, and past and present scientists working on a variety of ecological projects. Thus, one must wonder if, or how, these structures influence lemming population dynamics. For example, does the boardwalk along the Barrow Environmental Observatory (BEO) provide safe haven for lemmings and influence survival and reproduction? The fact is, the boardwalk blocks wind-driven snow and allows for snow to accumulate and be retained for longer periods than normal spring melt. In turn, this could provide cover and enhance survival for lemmings in the subnivean space. On the other hand, weasels, foxes, and Snowy Owls hunt along the boardwalk as there appear to be more lemmings there when the rest of the tundra melts off. The same can be said for snow fences and other large structures. This in turn could influence results of studies conducted adjacent to these man-made structures. Future lemming studies at Barrow should establish study sites distant from any human influences and in as unaltered natural habitat as possible.

## 10. Conclusions

1. The recognition by native people, explorers, and settlers of periodic mass outbreaks or plagues of lemmings dates > 100 years ago at Barrow (Murdock 1885 [39], Stone 1900 [40]) and hundreds of years worldwide (Stenseth and Ims 1993 [3]).
2. These casual observations were conceptualized by Collet (1895 [6]) as cycles and date back to the late 1800s.
3. The cycle concept was then championed in the 1920s by Elton (1942 [1]).
4. This review summarizes the literature between 1882 and 1974, relating to lemming cycles at Barrow, Alaska.
5. The cycle concept at Barrow, was buttressed from observations and studies from 1946 to 1974, but particularly 1955–1974 (Rausch 1950 [42], Thompson 1955 [34,44], Bee and Hall 1956 [45], Pitelka 1973 [27], Batzli et al., 1980 [48], Batzli 1981 [49], Pitelka and Batzli 1993 [28]).
6. Although an average time frame for peak populations did appear, defining a peak population as > or < than annual increases or decreases was arbitrary.
7. There were years when a peak lemming population might have occurred, but the amplitude was lower than the arbitrary pre-defined value of a peak versus a non-peak population.
8. Lemming populations at Barrow clearly fluctuated. But when an expected population high did not materialize, a qualifying statement was offered to explain the unexpected result and shape the cyclic concept back to regularity. For example, predation, food, weather, or immigration were used as reasons why the cycle was not on schedule.
9. Other researchers at Barrow also suggested lemming population peaks (Maher 1970 [63], MacLean et al., 1974 [64]) in different years, but these observations were not considered by Pitelka, even though they might have occurred (see Figure 1).
10. During the period from 1955 to 1974, only periodically did lemmings reach high populations. Depending on the data used and the analysis, intervals between lemming population highs could be 2–6 years, with an average of 3.8 years for data included in Pitelka (1973 [27]) or a range of 2–4, 2–6, 3–4, 5–6, years for data in Pitelka (1973 [27]), Pitelka and Batzli (1993 [28]).
11. The s-index used by Pitelka and Batzli (1993 [28]) was inferred to suggest a cyclic population. However, the s-index calculated from the Barrow studies appears to suggest population fluctuations within and between years, and not cycles (D. Holt, this review). And Krebs (p. 10, 2013 [5]) suggested the s-index measures population variability, not cyclicity.
12. Seventy-five years ago, Palmgren (1949 [108]) was one of the first to challenge the cycle concept, followed by others, Cole (1951 [109], 1954 [110]), Errington (1957 [111]), Anderson (1980 [112]), Garsd and Howard (1981 [113]). They wondered if cycles were just periodic rhythms or merely natural fluctuations in populations over time.
13. Kerlinger et al. (1985 [66]) specifically looked at lemming cycles and Snowy Owl winter irruptions in North America. They found no evidence for such an association, suggesting lemming cycles may not be regular events, but patterns in Snowy Owl numbers did occur.
14. The Barrow data clearly show lemming population fluctuations over time. However, researchers at Barrow used numerous qualifying statements to maintain the cyclic concept when natural events did not match expectations. The fact is, intervals between population highs were not equally spaced in time to support the true definition of a cycle. Furthermore, annual amplitude of lemmings is variable and never the same from year to year. If amplitude is variable, then density per unit area from year to year must be variable. This variability confounds the term cycle.
15. Perhaps the Barrow cycles were just periodic population fluctuations. The population reached a noticeably high amplitude in some years, but remained low amplitude in most years. And over time, these fluctuations resembled patterns but not cycles.
16. What does the word cycle really mean? Perhaps it is time to consider a better word, words, or no definition at all. The word cycle should be replaced with the phrase population fluctuations. Even Charles J Krebs (Krebs 1996 [54], Krebs 2011 [56], Krebs p. 192, 2013 [5]), one of the world’s experts on small mammal population dynamics, softened on the word cycle and advocated for population fluctuations.
17. The right conditions for producing high lemming populations occur only periodically, with each year linked, yet perhaps independent of pre- and post-year population highs. Thus, perhaps it would be better to just report annual lemming indexing results and compare years independently, versus setting definitions. Patterns can still emerge without having to assign a specific designation such as the four phases of a cycle (increase, peak, decline, low), that are not always sequential.
18. Maybe we have answered the population cycle question at Barrow. It is a combination of many factors (food, genetics, predation, physiology, snow conditions, social life, weather, among others), and it is not predictable. In any given year, one factor may influence events more than another and act independently. Similarly, in nature, a banner crop year, such as berry or mast production, only occurs periodically, and multiple factors must align for this to occur.
19. If we keep using the word cycle and its associated phases (increase, peak, decline, low), then we intuitively expect it to occur on schedule. And when it does not, we look for specific events to explain disruption in the cycle, other than natural population fluctuations.
20. Winter studies are encouraged, but they must be well-designed and long-term investigations. It is also conceivable that lemmings may breed every year under the snow with varying reproductive output. Perhaps lemmings surface more often in winter than previously believed. How else would some avian and mammalian predators survive when the tundra is snow-covered during spring?
21. If most studies are conducted adjacent to and within human-altered habitats, then are the results biased and not a true measure of natural processes?
22. I believe this historical to present-day review will assist the Iñupiat Eskimo people who own the lands to make informed decisions as the Barrow community expands and develops the adjacent tundra. This review will also provide information for federal and state managers and researchers to make informed decisions related to lemmings, Snowy Owls, Eider ducks, and other species of birds and mammals of concern in the Barrow region.
23. Overall Pitelka and colleagues used deductive reasoning to maintain the concept of a cycle, when in fact the data did not always agree. When the cycle was out of rhythm, Pitelka and others explained or inferred that it would have been cyclic had not some natural event occurred. However, all events I see in these studies were natural, and that is part of the lemming life history. The suggestion that cycles can occur 2–6 years or 3–4 years leads me to question the term itself. Furthermore, even some of Pitelka’s colleagues and students were unsure of what the peak population was in the cycle. Indeed, the definition was ambiguous. Furthermore, some researchers believed 1952 and 1953, 1964 and 1965, and 1971 and 1972 were back-to-back population peaks, but were not considered because of issues defining a peak. There were also other years when researchers were unsure what was a peak year versus a non-peak year (Figure 1). And what of the only collared lemming peak of 1971? The s-index suggests this is a cyclic population. So, does that mean collared lemmings only peak every 19 years at Barrow?

It is my conclusion that each year has its own signature and each year should be looked at independently over time, without definitions of a population peak or population low, and so forth. Then, over time, one can see population fluctuations without having to qualify pre-conceived values.

## Figures and Tables

**Table 1:** Density estimates of brown lemmings from Barrow, Alaska. Most trapping started in early June. There is no data for 1964. * MacLean 1974 [64] used lemming nests as an indication of density.

Source	Acre	Hectare	Notes
Bee and Hall 1956 [45]	50	125	optimal habitat
Bee and Hall 1956 [45]	20	50	other habitat
Thompson 1955 [34]	1	2	construction camp
Thompson 1955 [34]	42	5	local Tundra
Pitelka 1973 [27]	125	312	June 1960
Pitelka 1973 [27]	50	125	August 1960
Pitelka 1973 [27]	0.50	~1	June 1961
Pitelka 1973 [27]	1–10	2/3–25	June 1962
Pitelka 1973 [27]	50	125	June 1963
Pitelka 1973 [27]	1–5	~2/3–12/13	August 1963
Pitelka 1973 [27]	50–75	125–185	June 1965
Pitelka 1973 [27]	no data	0–100/0.4	cyclic peak 1950–1965
Pitelka 1973 [27]	no data	1/4.0	cyclic low 1950–1965
Maher 1970 [63]	70–80	175–200	cyclic peak 1953
Maher 1970 [63]	40–50	100–125	cyclic peak 1956
Maher 1970 [63]	70–80	175–200	cyclic peak 1960
Maher 1970 [63]	~5	12/13	cyclic lows—no data
Batzli et al., 1980 [48]	no data	0.02–225 ha^−1^	density equation 1955–1974
* MacLean et al., 1974 [64]	no data	0/ha	1969/1970
* MacLean et al., 1974 [64]	no data	42 ha^−1^	1970/1971
* Maclean et al., 1974 [64]	no data	27.5 ha^−1^	1971/1972
* MacLean et al., 1974 [64]	no data	14.4 ha^−1^	1972/1973

## Data Availability

No new data were created or analyzed in this study.
